# Ethics and the Future of Meaningful Work: Introduction to the Special Issue

**DOI:** 10.1007/s10551-023-05345-9

**Published:** 2023-04-17

**Authors:** Evgenia I. Lysova, Jennifer Tosti-Kharas, Christopher Michaelson, Luke Fletcher, Catherine Bailey, Peter McGhee

**Affiliations:** 1 Department of Management and Organization, Vrije Universiteit Amsterdam, De Boelelaan 1105, 1081HV Amsterdam, The Netherlands; 2 Babson College, Babson Park, MA USA; 3 University of St. Thomas, Minneapolis, MN USA; 4 University of Bath, Bath, UK; 5 King’s College London, London, UK; 6 Auckland University of Technology, Auckland, New Zealand

**Keywords:** Meaningful work, Business ethics, Future of work, Meaning in work, Technological unemployment, Artificial intelligence, Corporate social responsibility

## Abstract

The world of work over the past 3 years has been characterized by a great reset due to the COVID-19 pandemic, giving an even more central role to scholarly discussions of ethics and the future of work. Such discussions have the potential to inform whether, when, and which work is viewed and experienced as meaningful. Yet, thus far, debates concerning ethics, meaningful work, and the future of work have largely pursued separate trajectories. Not only is bridging these research spheres important for the advancement of meaningful work as a field of study but doing so can potentially inform the organizations and societies of the future. In proposing this Special Issue, we were inspired to address these intersections, and we are grateful to have this platform for advancing an integrative conversation, together with the authors of the seven selected scholarly contributions. Each article in this issue takes a unique approach to addressing these topics, with some emphasizing ethics while others focus on the future aspects of meaningful work. Taken together, the papers indicate future research directions with regard to: (a) the meaning of meaningful work, (b) the future of meaningful work, and (c) how we can study the ethics of meaningful work in the future. We hope these insights will spark further relevant scholarly and practitioner conversations.

## Introduction: The Future Is Now

When we initially proposed the Special Issue topic, “Ethics and the Future of Meaningful Work,” to the *Journal of Business Ethics* in 2019, we were contemplating advances in, for example: the technological conditions of work (e.g., automation, artificial intelligence), the workplace environment (e.g., worker mobility; co-working arrangements; increasing insecurity and work intensity), workplace demographics (e.g., differences related to age, career stage, and life-stage; gender contrasts; and efforts around diversity, equity, and inclusion), and geographical shifts (e.g., economic power rebalancing and increasing urbanization). Each of these factors has unique implications for transforming the world of work and therefore the meaningful connection people have to that work and its associated ethical implications. For example, when people’s work becomes automated, do they lose a link to what made that work meaningful, or are they freed up to pursue more meaningful work? What is the moral responsibility of organizations to enable the pursuit of meaningfulness in the workplaces of the future?

At that time, we could not have known that a global pandemic was approaching that would radically upend almost overnight how, where, when, and—most crucially what work means—*why* we work. Two international conferences that we had designated as opportunities to “workshop” potential submissions to the special issue were postponed and replaced by one global question-and-answer Zoom call. The strong levels of interest we witnessed in participating in the online forum demonstrated that scholarship on meaningful work was undeterred by the pandemic, but it also showed us that there is no replacement for face-to-face conferences to support meaningful, sustained interactions.

While many people, including us, were theoretically contemplating the implications of robots coming to take our jobs in an unknown, ambiguous, and uncertain “future of work,” a low-tech, old-style public health threat disrupted our present. The COVID-19 pandemic tragically claimed the lives of many people, including some family members and friends of (potential) contributors to and editors of the special issue. For survivors, it transformed—in both temporary and permanent ways—the world of work. The ensuing upheaval—including the designation of some workers as “essential”, increasing numbers of people working from home, and the so-called “Great Resignation” with the ensuing labor shortage—all have deep implications for employees, organizations, and even societies in terms of what work is offered, pursued, and further developed (e.g., Akkermans et al., 2020; Cook, 2021; Malhotra, 2021). The continuing effects of the COVID-19 pandemic further complicate our ability to experience, sustain, and provide meaningful work (e.g., Kramer & Kramer, 2020). Of course, the pandemic was not playing out in isolation. Other global issues—from political polarization, racial unrest, and the invasion of Ukraine by Russia, to the rapidly-advancing changes to the climate—compounded the instability of our economic, social, and governmental systems. Such macro-level turbulence and political unrest also makes people question their own sense of meaning and purpose, and creates a more uncertain future in which to understand and embed meaningful work (Fletcher & Schofield, 2021; Michaelson & Tosti-Kharas, 2020). The lesson, we believe—in addition to requiring us to stay nimble in our research, just as we have had to in our work lives these past several years—is that questions addressing the intersection of meaningful work, ethics, and the future of work, writ large, will become ever-more important as we collectively navigate a complex environment.

Even prior to the events of the last several years, research on meaningful work was on the rise. We witnessed a “Do what you love” cultural zeitgeist, as well as its ensuing backlash –which explored, for example, the ways in which the love employees feel for their work could be co-opted by organizations seeking to profit from and exploit this love (e.g., Cech, 2021; Jaffe, 2021; Tokumitsu, 2015). Management researchers also spent a great deal of time researching and cataloging what it means to view work as meaningful, including conceptualizations of work as a calling, passion, or purpose. Within the past few years, researchers have published several literature reviews (Bailey et al., 2019b; Blustein et al., 2023; Laaser & Karlsson, 2022; Lysova et al., 2019a; Thompson & Bunderson, 2019) and meta-analyses (Allan et al., 2019a, 2019b; Dobrow et al., 2023), an edited handbook (Yeoman et al., 2019), and three journal special issues (Bailey et al., 2019a; Laaser, 2022; Lysova et al., 2019b) on these topics, illustrating growing scholarly attention. Meaningful work was also the inspiration for the theme of the 2016 Academy of Management conference in Anaheim, which aimed to foster conversations about “Making Organizations Meaningful.” These developments signal not only increasing interest in the concept of meaningful work but also raise several significant and as yet unanswered ethical questions that would benefit from interdisciplinary attention.

By joining the dialogue between ethics and organization studies about meaningful work (e.g., Michaelson et al., 2014) with the ever-present reality of future trends in working, this Special Issue addresses a topic of wide-ranging interest and applicability. The papers in this Special Issue demonstrate that meaningful work is not only a managerial imperative to attract and retain future workers. It is also potentially a moral imperative of individuals to pursue a positive impact through their work; of organizations to provide work that serves a worthwhile purpose; and of societies to protect activities, including work, that give shape and significance to our lives. Before we turn to an overview of the Special Issue, the papers it includes, and the themes that it covers, we briefly review the existing literature on meaningful work, business ethics, and the future of work.

## What Do We Know About the Intersection of Meaningful Work, Business Ethics, and the Future of Work?

Business ethics is a discipline, meaningful work is a construct, and the future of work is a research setting or context. In theory, this means that they should be able to inform each other and do so without redundancy. Moreover, the future of work provides a kind of “extreme” research setting (Bamberger & Pratt, 2010) in which issues surrounding the ethics of meaningful work—from who has a right to it, to who has a duty to provide it, to what it means when meaningless work prevails—are heightened. There have been very few studies to date that truly speak to the intersection of these three topics–notable exceptions include Bowie, 2019; Kim & Scheller-Wolf, 2019; Smids et al., 2020; Turja et al., 2022. Therefore, we start by discussing prior research at the nexus between each of the pairs of topics in order to see what important questions remain to be addressed.

### Meaningful Work and Business Ethics

Although scholars rarely agree on a precise definition of meaningful work (Bailey et al., 2019b), the term generally refers to work that is personally and/or socially significant and worthwhile (Lysova et al., 2019a; Pratt & Ashforth, 2003). Part of the disagreement on the definition is due to the sheer number of disciplinary approaches and perspectives that have been used to study meaningful work (Bailey et al., 2019b). Historically, social scientists (more specifically, organizational psychologists) and philosophers (more specifically, business ethicists) have each studied meaningful work without a full awareness of each other’s perspectives (Michaelson et al., 2014). The former group has studied meaningful work as an individually fulfilling aspiration that has “positive valence” (Rosso et al., 2010, p. 95), clarifies one’s identity in terms of what one does and where one belongs (Pratt & Ashforth, 2003), and enables self-actualization and the sense that one’s work is worthy (Lepisto & Pratt, 2017). The latter have studied it as, among other things, a human right (e.g., Bowie, 1998; Schwartz, 1982), a virtue (e.g., Beadle & Knight, 2012), and a “fundamental human need” (e.g., Yeoman, 2014, p. 235). Meaningful work in organization studies has tended to draw primarily from the social scientific perspective to be framed in terms of an individual’s aspiration or motivation to perform meaningful work or an organization’s potential to perform better by providing opportunities for their employees to experience meaningful work (e.g., Bailey et al., 2019b; Lysova et al., 2019a). In business ethics, however, the focus has been more on the philosophical side, specifically on the responsibility of organizations or the state to foster a work environment in which the prevailing conditions preserve an individual’s autonomy to choose meaningful work (Michaelson, 2021; Michaelson et al., 2014). Of course, these perspectives are not mutually exclusive, and an interdisciplinary viewpoint opens up the possibility that responsible employers can serve individual employees’ preferences (e.g., Gifford & Bailey, 2018).

Meanwhile, an additional strand of social scientific research that explores work orientation, and particularly work as a calling—work that a person views as morally, personally, and socially significant and the end in itself (Wrzesniewski, 2012). However, there is extensive debate among both social scientists (e.g., Thompson & Bunderson, 2019; Dik & Shimizu, 2019) and philosophers (e.g., Care, 1984) about whether meaningful work that is a calling must focus on “serving self or serving others” (Michaelson & Tosti-Kharas, 2019, p. 19). Although philosophers have long debated whether meaningfulness is a subjective phenomenon, determined by the individual, or an objective phenomenon with clear references to moral conditions in which work is performed (e.g., Wolf, 2010). At the same time, meaningful work scholars have considered the well-being of others as a dimension of what makes work meaningful (e.g., Lips-Wiersma & Morris, 2009; Rosso et al., 2010). Yet, with a few exceptions, scholarship has rarely asked whether meaningful work *must* be good for others beyond ourselves (e.g., Michaelson, 2021; Veltman, 2016). The connection between meaningful work and business ethics has been also reflected in the growing research that explores how the experience of meaningful work is enabled by ethical and responsible leadership (e.g., Demitras et al., 2017; Wang & Xu, 2019; Lips-Wiersma et al., 2020) and corporate social responsibility (e.g., Aguinis & Glavas, 2019; Brieger et al., 2020; Janssen et al., 2022).

### Meaningful Work and the Future of Work

As of this writing, a Google Scholar search of the phrase “future of work” yielded nearly 90,000 results, about one-third of which were published within the past 5 years, and a regular Google search turned up 134 million hits. This observation clearly points to the recent exponential growth of interest in this topic. From the perspective of meaningful work, the future of work research tends to focus on whether job loss created by shifts like artificial intelligence and automation will pose a threat or opportunity to the quality of our work and lives, and more specifically to the pursuit of meaningful work. On the one hand, if jobs that are routine and potentially meaningless are automated, people could be freed up to pursue meaningful endeavors elsewhere, in their work and/or lives outside of work (e.g., Smids et al., 2020). This version of reality seems closest to the prediction by John Maynard Keynes nearly a century ago when he coined the term “technological unemployment” (1930/2010). As scholars, we should be careful about judging which jobs could be automated with little worker pushback in view of the highly personal experience of work as meaningful. A poignant example is a long-haul truck driver interviewed in the documentary, *The Future of Work and Death* (Blacknell & Walsh, 2016) who, when asked what his plans are should self-driving trucks render him redundant, replies, “I’m going to retire when I die in the truck.” Truck driving reported the second-highest rate of on-the-job deaths in 2020 according to the U.S. Bureau of Labor Statistics (2020); however, this example suggests that there are real tradeoffs between subjective and normative perspectives on what work *should* be automated. In other words, should certain jobs be saved to satisfy the subjective preferences of those who want to perform them, should the market decide, or should the state paternalistically prescribe which jobs are worth saving?

When we refer to the future of work, we should also be careful not to limit our understanding to only the implications of technological unemployment, specifically the headline-catching notion of sentient robots or machines taking over individual jobs. Recent reviews of the future of work identify several dimensions, including technological, social/demographic, economic, and political/institutional (Balliester & Elsheikhi, 2018; Santana & Cobo, 2020). Within this framework, automation and AI represent only one sub-category within the *technological* dimension, which also includes the emergence of new forms of work (e.g., gig work, platform work, telework), digitalization, and innovation. Indeed, there has been some relevant research that focused on studying experiences of meaningful work in new forms of employment like the gig economy (e.g., Kost et al., 2018; Nemkova et al., 2019; Wong et al., 2020).

The *social/demographic* dimension of the future of work includes issues affecting individual workers, such as burnout, work-nonwork conflict; attention to broader societal imperatives including corporate social responsibility; and issues affecting vulnerable workers, such as immigrants, minorities, and older workers. Here, research has addressed how workers respond to challenges to maintaining meaningful work, including the burnout and overwork that accompany the connection to broader social imperatives like animal welfare (Bunderson & Thompson, 2009; Schabram & Maitlis, 2017) or volunteer work (e.g., Toraldo et al., 2019). Some emerging research has started to explore how different groups, such as those in blue-collar work or from minoritized groups, may have restricted opportunities to access some forms of meaningful work (e.g., Allan et al., 2019a, 2019b; Lips-Wiersma et al., 2016).

The other two dimensions are the *economic*, which considers wage inequality, (un)employment, and job precarity, and the *political/institutional*, which considers industrial relations, trade unions, and the labor market. These future of work dimensions are addressed in sociological (e.g., Gallie, 2019; Lasser & Karlsson, 2022) and vocational research (e.g., Allan et al., 2020; Allan et al., 2019a, 2019b; Duffy et al., 2016) on decent work, or the work that provides access to adequate healthcare, protection from physical and psychosocial harm, adequate compensation, adequate rest, and organizational values that complement family and social values. Research that explores decent work as a psychological work experience builds on the assumption that economic constraints and marginalization limit the possibility for individuals to secure decent work, and, therefore, for individuals to experience meaningful work (Allan et al., 2019a, 2019b; Duffy et al., 2016). A review of the literature reveals that research on meaningful work has mostly considered the technological and social/demographic dimensions, viewing them as presenting both future threats and opportunities, but has paid less attention to the economic and political/institutional dimensions.

### Business Ethics and the Future of Work

It might be fair to say that any business ethics research that is concerned about employee well-being is focused on making the future of work better than the present. From Adam Smith’s worry that division of labor capitalism made workers “stupid” to Karl Marx’s concerns about the alienation of labor, ethicists and political theorists have tried to envision better workplaces that respect human dignity (e.g., Pirson, 2017), support employee participation in workplace governance (e.g., Hsieh, 2005), and align with employees’ ethical values (e.g., Paine, 2004). Much business ethics research has been mobilized by the role of markets in fostering economic inequality (e.g., Beal & Astakhova, 2017), unequal treatment of workers in different jurisdictions (e.g., Donaldson & Dunfee, 1999), and structural injustices exacerbated by the COVID-19 pandemic (e.g., Van Buren & Schrempf-Stirling, 2022). Generally, business ethicists have worried more about sweatshop abuse (e.g., Arnold & Bowie, 2003), overwork (e.g., Golden, 2009), and workaholism (e.g., Boje & Tyler, 2009) than they have worried about technological future in which there is no work at all.

More recently, perhaps as robots and artificially intelligent machines have become more of a reality, scholars have begun to fret more about technology, particularly whether life will be “worth living in a world without work” (Danaher, 2017, p. 41). It is possible that the jobs people lose to technology will be ones that were meaningful to them, making it difficult to move on (e.g., Smids et al., 2020; Turja et al., 2022). While the foreseeable future probably portends continued concern about familiar ethical challenges at work, the unforeseeable future could possibly bring unfamiliar ethical challenges having more to do with the “axiological challenge” (Kim & Scheller-Wolf, 2019, p. 320) of how to replace work in a meaningful life. As long as human work remains, however, technology will influence the freedom and autonomy that have the potential to make work worth doing. Already, algorithmic decision-making about human resources (e.g., Leicht-Deobald et al., 2019) and the technological monitoring of employee activity (Martin et al., 2019) are seen to infringe upon employee autonomy, a potential invasion of the right to privacy as well as a critical element of the experience of meaningful work.

## What Are the Insights From This Special Issue?

Our quest in this Special Issue was to seek to research the relatively understudied intersection of all three areas: business ethics, meaningful work, and the future of work. The call for papers ultimately attracted 72 submissions, a strong response in terms of both quantity and quality that we believe further justifies the novelty, timeliness, and resonance of the topic. After several rounds of peer-reviewed revisions, seven papers were finally selected to comprise this Special Issue along with this introductory essay. This Special Issue itself represents a variety of contributions highlighting the diversity of its authors. It features both conceptual and empirical contributions, as well as philosophical and social scientific approaches. Its authors come from universities in Australia, Canada, Denmark, Finland, South Africa, Sweden, Thailand, the UK, and the US. It also seems important to note, although this was not by design, that its editors come from universities in the Netherlands, New Zealand, the UK, and the US and represent multiple disciplinary approaches, including social science/psychology and philosophy/ethics. Overall, we hoped this Special Issue would be a valuable opportunity to truly bridge these divergent viewpoints to advance this constructive dialogue. Below, we provide a brief overview of each of the papers included in this issue.

First, Sarah Bankins and Paul Formosa (this issue) offer a conceptual article, *The ethical implications of artificial intelligence (AI) for meaningful work*. The authors note that, since the industrial revolution, technological advancements have always had significant implications for meaningfulness through changes to work, skills, and affective experiences. In the paper, they propose three paths of AI deployment and discuss how, through each of these paths, AI may enhance or diminish five dimensions of meaningful work–task integrity, skill cultivation, and use, autonomy, task significance, and belongingness. The paths they identify are: *replacing*, in which AI takes on some tasks while workers remain engaged in other work processes; *tending the machine*, in which AI creates new forms of human work; and *amplifying*, in which AI assists workers with their tasks and/or enhances their abilities. Bankins and Formosa argue that while some pathways, such as tending the machine, may significantly limit opportunities for meaningful work, AI has the potential to make some types of work more meaningful by undertaking more boring tasks and thereby amplifying human capabilities. The authors offer the caveat that, while some jobs may be affected by just one of these paths, others may experience all three. Bankins and Formosa further draw on the “AI4People” ethical AI framework, which identifies five principles–beneficence, non-maleficence, autonomy, justice, and explicability–that enable us to assess the ethical implications of AI implementation on meaningful work. In summary, their argument suggests that there is not likely to be one single impact of AI on meaningful work, as it has the potential to make work meaningful for some workers but it can also make work less meaningful for others.

Next, in their article, *Saving the world? How CSR practitioners live their calling by constructing different types of purpose in three occupational stages*, Enrico Fontana, Sanne Frandsen, and Mette Morsing (this issue) examine one form of deeply meaningful work, work as a calling, to address the question of whether the sense of purpose that underpins a calling changes over time. In particular, they challenge the notion that callings constitute the end point of a journey towards self-actualization and instead draw attention to the tensions experienced by individuals as they seek to pursue their callings within organizational settings whose agendas may be very different from their own. Such tensions may be especially acute for Corporate Social Responsibility (CSR) practitioners who may be driven by altruistic goals that potentially contrast with the realities of organizational life. Through a qualitative investigation of CSR practitioners in Sweden, the authors find that respondents constructed the purpose of their work differently across career stages. Early-career practitioners pursued an *activist* purpose aimed at affecting transformational change in their company’s approach to CSR, while mid-career practitioners constructed a *win–win* purpose grounded in the sense that not only did their work tackle social issues but it also supported their employer’s aims. Finally, late-career practitioners adopted a *corporate* purpose that prioritized corporate success unrelated to social aspirations—their own or their organization’s. This research reveals that callings can be lived out in different ways that may differ from a perception that callings are essentially ethical and prosocial. The authors highlight the potentially harmful long-term effects of companies prioritizing commercial goals over the social aspirations that drive CSR practitioners’ early work orientations.

In *Body-centric cycles of meaning deflation and inflation*, authors Anica Zeyen and Oana Branzei (this issue) draw on a longitudinal study of 24 self-employed disabled workers in UK organizations to explore how respondents used their bodies to make meaning at work during the COVID-19 global pandemic. In so doing, they employ an “ethics of embodiment” theoretical lens that views meaning-making in the context of work as inherently body-centric. Disabled workers are particularly relevant to study in this context given how marginalized they may be at work precisely because of their bodies, especially during the COVID-19 pandemic. Utilizing interview and diary data and analyzing it in an abductive fashion, the authors found that disabled workers purposefully enrolled their bodies in what they term *dramas of suffering* or *dramas of thriving*. These dramas not only rendered mind–body differences visible to the workers and others at work but also instigated their meaning-making at work: dramas of suffering deflated meaning while dramas of thriving inflated it. The authors developed a process model of meaning-making through body drama that showed this process to be cyclical: suffering demanded more meaning-making, which often entailed more suffering, and so on, with a similar cycle for thriving. Importantly, each respondent was locked into their own body-driven cycle of either suffering or thriving. This study provides a unique perspective on the various forms of worker disability, as well as the various paths disabled workers can take to strengthen or weaken meaningfulness in response to a challenge like the pandemic.

Frank Martela’s (this issue) paper, *The normative value of making a positive contribution—Benefiting others as a core dimension of meaningful work*, asserts that normative accounts of meaningful work should be expanded to include contributing–doing work that has a positive impact on the lives of other people—as another key moral dimension of meaningful work. While the view that work must make a social contribution may be assumed in some subjective accounts of meaningful work—such as when meaningfulness is associated with corporate social responsibility—it is not always viewed as a normative requirement. Martela reflects on normative accounts of meaningful work that have been built mostly around autonomy, the capability for self-development, and the avoidance of alienation. What makes these accounts normative is that they tend to impose moral responsibilities on employers to provide work that preserves the freedom of the worker to pursue work that is subjectively meaningful. However, while these accounts may be good for the worker, Martela’s account of meaningful work may also impose a normative obligation on the worker to perform work that is good for *others*. Moreover, this account of meaningful work not only ascribes responsibilities to the individual, but it also implies that the employer is morally responsible for providing work that enables the individual to make a positive contribution to society. Martela argues that doing work that does not make a positive impact or being separated from the positive contribution one’s work is making, is its own form of alienation from work.

David Silver (this issue) echoes this theme in *Meaningful work and the purpose of the firm* while expanding on what the pursuit of a positive social contribution means for the organization. He argues that it is important to create products and services that provide a benefit to the people who ultimately use them. However, he asserts that doing so is the fundamental goal of the firm, not merely the aim of meaningful work. Silver thus situates his examination of meaningful work in the context of debates about the purpose of the firm, particularly against the backdrop of a profit maximization thesis that has only relatively recently been challenged as a legitimate organizational purpose. Because people typically work within organizations, Silver argues that employees’ opportunity to perform work that is meaningful by virtue of its societal contribution can either be supported or undermined by their employer’s purpose. He proposes that when that purpose is profit maximization alone, even work that makes a positive contribution to others, whether organizational shareholders or other stakeholders, may not feel meaningful. Conversely, when the purpose of the firm is to benefit end users, even work that might otherwise be viewed as meaningless can be imbued with a sense of serving a meaningful purpose.

In his paper, *What makes work meaningful?* Samuel Mortimer (this issue) acknowledges that meaningful work must be both personally motivating and objectively worth pursuing. He observes that in qualitative interview studies of workers who feel their work is deeply meaningful, they not only describe how the work makes them feel, but also explain how their work contributes to a broader purpose or mission to which they are committed. Mortimer argues that the answer to the question of what makes work meaningful can be found in a burgeoning philosophical consensus that there must be both subjective *and* objective elements to meaningful work. He argues that subjective, or experiential, accounts of meaningful work are of value to providers of work endeavoring to attract and retain employees but that it is circular to tell someone who is seeking meaningful work to find work that feels meaningful to them. However, Mortimer also recognizes that so-called objective accounts of meaningful work show that such work, which sometimes entails significant sacrifice, can have the unintended consequence of undermining the pursuit of a meaningful life. He aims to integrate these two accounts of meaningful work by arguing that volitional commitment to a particular line of work makes it normatively meaningful.

Santiago Mejia (this issue), in *The normative and cultural dimension of meaningful work: Technological unemployment as a cultural threat to a meaningful life*, also seeks to expand our understanding of meaningful work beyond the subjective experience of the individual worker. In doing so, he discusses not only what makes work meaningful but also why work matters. Mejia draws attention to the pervasiveness of work in our society, which is evident in the fact that conventional measures of societal well-being include, for example, the unemployment rate, how we identify ourselves with our occupations, and how we measure time in terms of pre-work education, our working life, and post-work retirement. Mejia imagines a future in which we may potentially work less, or not at all, and envisions a cultural crisis in which the absence of work will take away a central organizing *telos* around which our contemporary lives gravitate—including the opportunity that work provides to contribute to our own and the common good.

## What Are the Takeaways and Future Research Directions?

A consideration of the common themes emerging from the Special Issue papers points to three fundamental questions: (a) What is the meaning of meaningful work? (b) What is the future of meaningful work? and (c) How should we study the ethics of meaningful work in the future? We believe attention to these themes and questions would be particularly fruitful moving forward to inform the future of both research and practice on meaningful work, business ethics, and the future of work.

### What Is the Meaning of Meaningful Work?

One of the main themes identified in the Special Issue concerns how we conceptualize meaningful work. In fact, the four final papers together grapple with this question, while each author takes a distinct perspective on what “counts” as meaningful work. Martela (this issue) and Silver (this issue) argue that meaningful work must make a societal contribution. Conversely, Mortimer (this issue) and Mejia (this issue) merge the societal, normative view of meaningful work as doing good with the more personal and sociocultural view that meaningful work is volitional (Mortimer) and culturally determined (Mejia). Although the great variety of experiences of meaningful work makes it an endlessly fascinating concept, many philosophical questions remain, including: Is what unites all such experiences the subjective sentiment on the part of the worker that what they are doing is meaningful? Is it possible for them to be objectively mistaken—for the work one thought to be meaningful actually to be quite meaningless? Given that immoral work has been used as a counterexample to challenge meaningful work subjectivism, is it reasonable to suggest that all meaningful work must be moral work?

In sum, there remains room to articulate more clearly what “the search for something more” (Ciulla, 2000) in one’s work consists of, whether from the subjective or objective perspective. In their implicit dialogue with one another and with philosophical research on meaningful life (especially Wolf, 2010) and meaningful work (including Michaelson, 2021; Veltman, 2016), these four papers in the Special Issue by Martela, Silver, Mortimer, and Mejia all suggest that while the subjective experience of meaningfulness may be necessary to render work meaningful, it is not sufficient to render work *normatively* meaningful. In fact, to our knowledge, these papers comprise the most comprehensive, collective attempt to challenge the assumption, shared by many organization scholars and business ethicists, that what makes work meaningful is “in the eye of the beholder” (Michaelson et al., 2014, p. 86). They challenge this so-called subjectivist account of meaningful work by suggesting that meaningful work ought to be moral work, although some are careful to note that this does not necessarily imply that all normatively moral work is experienced as meaningful work (also see Yeoman, 2014).

We note that, in addition to these four papers, dozens more of the submissions we received questioned what meaningful work means. While achieving a consensus would increase continuity and foster a more coherent dialogue, a pluralist conceptualization of meaningful work permits the field to engage with a broader perspective on what constitutes meaningful work. We thus encourage future researchers to continue to conceptually and empirically explore the multiple meanings of meaningful work, similar to the efforts of research on calling (e.g., Dik & Shimizu, 2019; Thompson & Bunderson, 2019). In any case, scholars should specify upfront in their studies what they mean by meaningful work and why they have chosen this particular perspective. Furthermore, in line with Mejia’s (this issue) suggestion that meaningful work is culturally determined, we also call for cross-cultural research that examines how cultural accounts of what makes work worth doing enable or hinder people’s access to meaningful work. So far, research addressing these important questions remains scarce (cf. Boova et al., 2019). Moreover, we encourage scholars to consider how such debates about the meaning of meaningful work could shape whether and how organizations should implement certain organizational policies and practices with the aim of enabling meaningful work.

### What Is the Future of Meaningful Work?

To our surprise, almost none of the papers that were submitted to and eventually included in the Special Issue primarily used the future of work as a research focus. Instead, many authors raised issues related to the future of work in the discussion as a contextual factor that might complicate the findings. This may indicate a reluctance on the part of authors to dive into these issues, or a paucity of research to build upon. Only Bankins and Formosa (this issue) and Mejia (this issue) put the future of work front and center. Both papers address the technological aspect of the future of work, speaking to the potential for technology, if not managed correctly, to pose a fundamental threat to meaningful work. Two other papers in the Special Issue—Fontana, Frandsen, and Morsing (this issue) and Zeyen and Branzei (this issue)—address the social dimension of the future of work. We look forward to reading future research that considers the full spectrum of relevant and timely settings at the intersection of technological, social, political, and economic dimensions of the future of work. Also, scholars should explore the ways in which these various domains interact with each other to facilitate or constrain organizations’ ability to provide, and employees’ ability to access meaningful work. To spark such a research program, we invite meaningful work scholars to team up with scholars studying the implications of the future of work. This could also boost much-needed interdisciplinary research on meaningful work.

We note that the paper by Fontana, Frandsen, and Morsing (this issue) considers corporate social responsibility, an element of the social dimension of future work that has gained increased importance, as organizations take more ownership of their role in societal ills such as climate change. Similarly, Zeyen and Branzei (this issue) discuss how disabled workers adapted to new and future realities of working triggered by the COVID-19 pandemic. An important point that both papers highlight is the value of taking a process view of change over time, whether in exploring meaning-making at work (Zeyen & Branzei, this issue) or revealing deep experiences of meaningful work captured by the concept of work as a calling (Fontana, Frandsen, & Morsing, this issue). In this way, these papers emphasize the necessity for future research to consider how meaningful work changes and fluctuates over time, an issue that has so far received only scant attention. Qualitative studies (Bailey & Madden, 2017; Mitra & Buzzannell, 2017; Lips-Wiersma & Wright, 2009) have uncovered the temporal and tensional nature of meaningful work, while quantitative studies have found that meaningfulness fluctuates within a person on a weekly (e.g., Lysova et al., 2022), daily (Vogel et al., 2020), and situational (Fletcher et al., 2018) basis. We therefore call for future research to emphasize a temporal understanding not only of meaningful work itself but also of how we study it by examining, for example, how people respond to future threats to meaningful work, or how newcomers’ evaluations of meaningfulness fluctuate as they move in between onsite and remote work environments.

### How Should We Study the Ethics of Meaningful Work in the Future?

The articles included in the Special Issue reflect the philosophical as well as social scientific disciplinary approaches to studying meaningful work, making an important step toward bridging disciplinary boundaries and create a shared dialogue that moves the field forward. However, we cannot help but notice that within each paper, one disciplinary perspective remains dominant. Therefore, our first three papers, representing the social scientific perspective, best dialogue with each other, while the last four, representing the philosophical perspective, primarily speak to each other. To move the field forward, we hope that in the future scholars in different research disciplinary perspectives would continue engaging in a dialogue with each other.

We believe there are ample opportunities to consider interdisciplinary perspectives on meaningful work—including in theorizing and, where applicable, data analysis—ideally involving interdisciplinary co-author teams in the design of studies informed by multiple perspectives. Such studies could include novel research contexts or mixed methods, perhaps blending qualitative and quantitative empirical approaches as well as a focus on interventions—see Fletcher and Schofield (2021) and Lysova et al. (2022) as examples. We also call for research to address the multiple levels of analysis inherent in studying meaningful work—e.g., cultural and societal, industry and occupational, organizational, and individual levels—all of which may inform and/or contrast with each other.

One particular avenue to explore is the intersection between equality, diversity, and inclusion (ED&I) and meaningful work, which we see to be promising in terms of promoting much-needed theory development. ED&I is becoming increasingly critical to address across various literatures, particularly given likely future population and demographic shifts, the continued rise of populism and nationalistic agendas as well as war and political frictions between countries, and the economic inequalities and uncertainties arising in the post-pandemic world. This requires enriching the understanding of meaningful work for minoritized individuals that involves: contextualizing meaningful work within the wider landscape of their identity, their organizational and occupational setting; and their relationships at work (Fletcher & Beauregard, 2022; Fletcher & Everly, 2021; Zeyen & Branzei, this issue). However, this understanding also needs to examine current, and potential future, objective conditions of work which may compound disadvantages.

In conclusion, rather than being the ultimate word on ethics and the future of meaningful work, we view this Special Issue as an initial, hopefully, foundational step to better understand how these topics connect with each other. Since we as academics think about our own meaningful work, we hope that some of the suggestions we have made in this essay will help spur exciting new ideas and contributions. We also hope that some of the insights generated in these papers can inform real working people, work organizations, and people in positions to set organizational and public policy. Given how much the near future of work was transformed by COVID-19 and how many major changes loom on the horizon, it seems there will be no shortage of opportunities to reflect on what work means *now* and how meanings have changed and will change in the future, including whether work is seen as meaningful.
